# Transesophageal Echocardiogram: Still a Powerful Tool in the 21st Century for the Diagnosis of Aortic Dissection

**DOI:** 10.7759/cureus.34368

**Published:** 2023-01-30

**Authors:** Rui Flores, Joana Lopes, Vítor Hugo Pereira, Nuno Salomé

**Affiliations:** 1 Cardiology, Hospital de Braga, Braga, PRT; 2 Internal Medicine, Hospital de Braga, Braga, PRT

**Keywords:** cardiac magnetic resonance (cmr), trans-esophageal echocardiogram, transthoracic echocardiogram, type b aortic dissection, aortic disease

## Abstract

A 63-year-old woman with a history of previous anaphylactic reaction to iodate contrast presented with sudden back pain during exertion associated with elevated D-dimer levels. Transthoracic echocardiogram was unremarkable. She was unable to perform a computerized tomography for further evaluation of the aorta due to her allergic background. Transesophageal echocardiogram disclosed a type B aortic dissection. This case report recalls the importance of transesophageal echocardiography in the algorithm for diagnosing aortic dissection in scenarios where CT is not possible.

## Introduction

Aortic dissection corresponds to a form of an acute aortic syndrome associated with high morbidity and mortality [1-4]. Early diagnosis using imaging techniques is essential, and the gold standard for diagnosis is contrast-enhanced computerized tomography (CT) [1-4]. In rare cases of unavailability or impossibility of using CT, magnetic resonance imaging (MRI) and ultrasound can be useful [1-4]. Aortic dissection can be classified according to its location and extent, time of evolution, and vascular compromise of limb or viscera perfusion [1-6]. The Stanford classification subdivides the dissection according to the involvement of the ascending aorta [1,4]. Type A dissections, which comprise dissections involving the ascending aorta and/or the aortic root, are associated with lower survival and a higher risk of complications [1,2,4,6]. According to the time of evolution, the tearing can be divided into hyperacute (< 24 hours), acute (two to seven days), and chronic (> 30 days) dissection [1,4]. Perfusion of the limbs or viscera through the false lumen allows the distinction between complicated and uncomplicated dissections [1,2,4]. Any of these classifications has a therapeutic impact [1,3,5,7]. The main risk factors include hypertension, connective tissue diseases, bicuspid aortic valve, and a history of cardiac surgery [2-5,7].

We report a case of type B aortic dissection, the diagnosis of which required the use of transesophageal echocardiography (TEE).

## Case presentation

A 63-year-old Brazilian woman came to the Emergency Department due to sudden back pain during exertion. Her past medical history included untreated depression and dyslipidemia. She reported a family history of Marfan syndrome but denied having a formal diagnosis herself. In the course of her Marfan screening in Brazil, she developed a severe anaphylactic reaction to iodinated contrast while undergoing a thoracic contrast CT. Her diagnostic workup was suspended due to this allergic reaction and had not been resumed.

**Figure 1:**
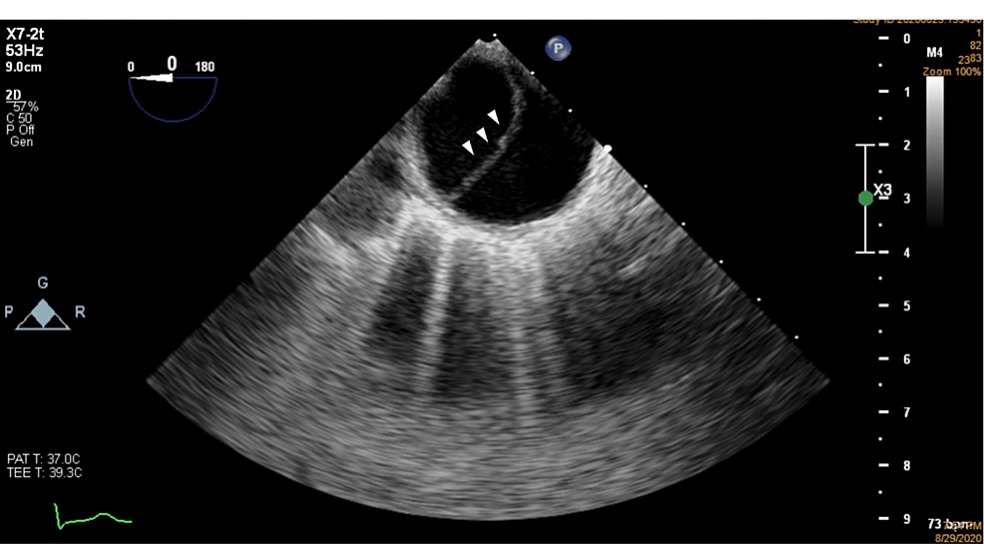
Aortic evaluation by TEE showing flap image (arrowhead) in the thoracic aorta distal to the emergence of the left subclavian artery. TEE: transesophageal echocardiography

At admission, she was hemodynamically stable and presented palpable and symmetric pulses in all four limbs. Her electrocardiogram was unremarkable, except for sinus tachycardia. Troponin levels were normal, but D-dimer levels were markedly elevated. The dimensions of the aorta appeared normal on the chest radiography (CXR). A transthoracic echocardiogram (TTE) was requested and it showed a normal-sized aortic root and ascending aorta. The left ventricle had normal dimensions and its function was normal. The right ventricle was also normal-sized and had normal function. Besides a mild aortic regurgitation, the remaining valves had normal function. No indirect signs of pulmonary embolism were seen. Due to the history of severe iodine allergy, the patient could not undergo a CT scan for further evaluation. She was proposed for MRI, which was not available at the time. A TEE was performed instead. The aortic root and ascending aorta were of normal morphology. Figure 1 and Videos 1-2 show a Type B aortic dissection starting at the level of the aortic arch and extending itself at least throughout the thoracic aorta. Obviously, the abdominal aorta could not be adequately evaluated through this exam. 

**Video 1:** Descending aorta by TEE. The aortic dissection flap separates the true and false lumen. The true lumen is smaller due to compression by the false lumen. TEE: transesophageal echocardiography

**Video 2:** Aortic dissection by TEE. Evaluation of type B aortic dissection using color Doppler. The smaller cavity, which is filled by the color Doppler, corresponds to the true lumen. TEE: transesophageal echocardiography

**Figure 2:**
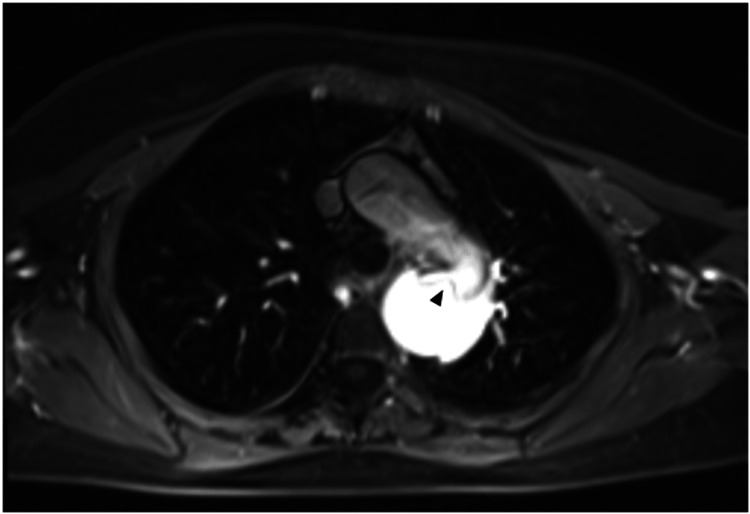
MRI with gadolinium enhancement showing a flap (arrowhead) at the beginning of the descending aorta.

**Figure 3:**
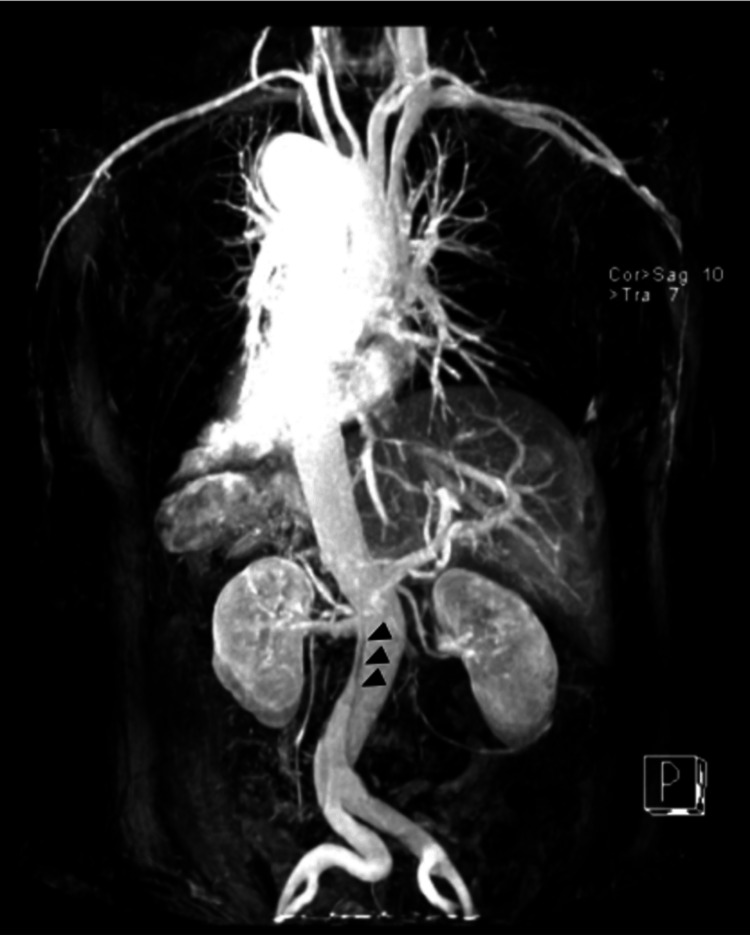
Three-dimensional MRI reconstruction of the aorta showing a flap (arrowhead).

After placing an arterial line for invasive hemodynamic evaluation and a central venous catheter, the patient started esmolol and sodium nitroprusside infusions and was transferred to another hospital for vascular surgery evaluation. An MRI was then performed to assess the extent of the dissection and the vascular compromise of the viscera (Figures 2-3; Videos 3-4).

**Video 3:** Abdominal MRI with gadolinium enhancement. MRI sequence imaging showing the extension of the aortic dissection.

**Video 4:** MRI sequence of the entrance tear of the aortic dissection.

MRI confirmed a type B aortic dissection in a normal-sized aorta with an entrance tear by the descending aorta and extending throughout the thoracic aorta, abdominal aorta, and common iliac arteries, without significantly compromising the lumen of splanchnic vessels. The diagnosis of uncomplicated type B aortic dissection was made and the patient maintained conservative treatment with beta-blockers and systemic vasodilators. The hospitalization period was uneventful and the patient was discharged after two weeks. The study of connective tissue diseases was inconclusive.

## Discussion

An aortic dissection is a subtype of aortic syndrome with an incidence of 15 per 100.000 people, which is associated with a high rate of morbidity and mortality [1-4]. It occurs by tearing the intima of the vessel, which allows the passage of some blood from the true lumen of the vessel to a virtual space (or false lumen) that propagates anterogradely or retrogradely [1-3].

The diagnosis requires a low threshold of suspicion in the presence of chest, back, or abdominal pain [1,2,4,8]. The gold standard of diagnosis is a CT, which allows the identification of an intimal flap dividing the aortic lumen into two portions (the false and true lumen) [1,3,4,8]. Other imaging tests, like a TTE, TEE, or MRI, can also be used when CT does not assure the diagnosis or cannot be used [1,2,4,8,9]. Some rare contraindications to performing a CT include severe allergic reaction to iodinated contrast, hemodynamic instability, acute heart failure, or inability to cooperate [1,7]. The treatment of aortic dissection can be surgical, endovascular, or conservative with medical therapy [1,3,5,7-9]. In most cases of uncomplicated type B dissection, treatment can be conservative using negative chronotropic drugs associated with systemic vasodilators, thus decreasing the likelihood of tear propagation and complications [1,3,5,7,9]. Endovascular or surgical treatments are kept for complicated dissections or subacute to chronic stages of the disease [1,3,5,7-9].

We report a very peculiar case of type B aortic dissection in a patient with a morphologically normal aorta and without a formal diagnosis of connective tissue disease. The diagnosis was challenging due to the limitations imposed by the severe allergic reaction to iodine. Despite having a lower diagnostic sensitivity, the use of TEE was essential in a hospital without 24-hour MRI availability [1,2,4]. This case highlights the importance of never forgetting second-line complementary exams in the diagnosis of potentially life-threatening pathologies.

## Conclusions

Aortic dissection is an easy diagnosis to miss if we do not have a high clinical suspicion. It is associated with high morbidity and mortality and we must be aware of all possible diagnostic tests. We report the case of a patient unable to perform a CT for the diagnosis of a type B dissection, in which the use of TEE was essential. This case highlights the importance of never forgetting second-line complementary exams in the diagnosis of potentially life-threatening pathologies.
